# On the Treatment of Pulpless Teeth

**Published:** 1884-02

**Authors:** Y. H. Balkwill


### On the Treatment of Pulpless Teeth.

BY Y. H. BALKWILL, L.D.S. ENG.

MR. PRESIDENT AND GENTLEMEN.—I am not going to pretend to advance any new doctrines or practice in treating pulpless teeth; but a rational treatment of them has been so recently established that a record of the most obvious facts in connection with it, if drawn from experience, may still be considered not entirely useless.

We can many of us remember the time when to fill a pulpless tooth was an adventure entered upon in fear and trembling, but too often justified by results. Then came a time when the operation of Rhizodontrophy was hailed as a great boon, and many were the ingenious devices for making a plug with a drainage tube through it. This method often resulted in a useful tooth for several years, yet leaving access of oral fluids to the pulp cavity, was not satisfactory. But it was not until Mr. Lister broached his doctrine of antiseptic surgery that any correct treatment of pulpless teeth was followed. Methods were from time to time described, which, looking back from what we now know, we can see approximated to the modern treatment, but still the treatment was felt to be empirical, and success was looked upon more in the light of a personal triumph than as the ordinary result of orthodox and intelligent practice.

Since the advent of antiseptic surgery all this has been changed, and I may safely say that no branch of the medical profession was quicker to recognize and adopt into practice, or has now a profounder conviction of the soundness of antiseptic surgery, than the dental profession.

I wish to describe a few typical cases, not in any way as statistics, but from having had them a long time under view they may be of value as illustrating details of practice.

Teeth having lost their pulps, which may require treatment at our hands, may be divided into three heads.

1st. Those whose pulps we have wholly or partially removed, generally after the application of arsenic.

2d. Those in which the pulp has disappeared before the tooth came to us for treatment, but where there is no sign of dental periostitis.

3d. Where there is, in conjunction with the loss of the pulp, more or less of inflammation of periosteum, ranging from a slight tenderness on percussion to alveolar abscess.

The question arises in connection with the first class: Whether it is always necessary to entirely destroy sensibility in the whole of the pulp.

My own opinion is, that after we have by candor discharged our minds from the suspicion of being biassed in judgment by the occasional difficulty of the operation, there still remains a very serious doubt as to this necessity.

I will relate a case which much impressed me. About eight years ago I applied arsenic to the pulp of an upper molar for a lady, and on attempting to remove the pulp it was found that about one third of the apical part was still so sensitive that any attempt at removal was negatived. As she was leaving Plymouth the next morning, I determined upon filling over the remains of the pulp. I do not know if I am right in my therapeutics, but I think I have observed that arsenic acts partly by paralyzing the vaso-motor nerves, so that irritation of the pulp after the application of arsenic no longer produces any increase of vascularity and consequent danger of inflammation. I placed a layer of carbolized wool, to be described a little further on, over the sensitive part of the pulp, and filled the remainder of the tooth with Sullivan's cement. The tooth is still in the head and has given rise to no gum-boil or pain since, or at least it had not a few months ago.

The success of this case encouraged me to others, and I now feel little hesitation in leaving part of the pulp in the root canals after the application of arsenic when circumstances indicate it.

I have to record a few failures in which the teeth have remained tender and sensitive to heat or cold and have had to be restopped, but these are practically very few.

The second class of teeth are those in which the pulp is absent, and there is no evidence of pericemental irritation; these used to be regarded as teeth of especial suspicion. The conditions of danger are, I think, pretty generally recognized. The pulp chamber is occupied by a fluid in a state of putrescent fermentation, as sufficiently evidenced by its smell, with the presence of bacteria. If any of the putrescent fluid is retained in the apices of the pulp cavity when the tooth is stopped, matter is formed in the periosteum commencing at these apices, which, having no outlet, forms an abscess with the attending inflammation. It has been often stated in our current literature that decompostion going on in the putrescent fluid, liberates gas, which, escaping by the apical foramen, adds to, if it does not excite, the inflammation. I was under this impression myself, but having performed the following experiment to verify the facts, am somewhat staggered in this opinion.

I took two teeth, extracted for alveolar abscess, having putrescent pulp cavities, cleaned out the cavities of decay, without disturbing the pulp cavities, and filled them up with gutta percha to act as a cork, and then coated the teeth, all but the apices of the root, with melted resin and wax. These were then placed roots uppermost in the bottoms of two jam pots full of water which had been boiled, and two small wide-mouth bottles full of water inverted over them; it will be clear that any bubbles arising from the foramina at the apices of the roots would be retained by the bottles; but up to the present time none have appeared. The bottles were placed over the teeth on July 30th, and kept in the upper part of a greenhouse for the sake of warmth. I will now pass them around for inspection.*

The difficulty in practice is to clean and disinfect the extremity of the canal, and to do this I find crystals of permanganate of potash extremely useful. This is easily conveyed into the pulp cavity, and is extremely soluble and penetrating—of which

*This was written on August 21st; the two following days were very warm, and when the paper was read, and the experiment exhibited, two extremely small bubbles were just perceptible in each bottle.

I will mention an instance more convincing than gratifying. In treating an upper cuspid for a gentleman, I had occasion to break off in the middle of the operation for a short time, so placing a small crystal of permanganate of potash in the pulp cavity, and stopping it with a piece of gutta percha to keep it from staining the crown of the tooth through the cavity of decay, I requested him to return in a couple of hours, which he did. I then found to my dismay that the permanganate had permeated all the dentine of the root and stained it a fine black; as some of the root was exposed, this was mortifying, but at the same time it convinced me of the extreme penetrability of permanganate of potash. I may add that this case happened nearly ten years ago. I saw the gentleman last week. There is still a slight indication of staining in the root, but the tooth is firm and without tenderness or gumboil.

My practice is, after syringing out the pulp cavity as well as I can, to put in a small crystal of the permanganate, break and churn this up with a canal probe, and let it remain about five minutes, then syringe out until the water runs clear. Wipe out pulp cavity and dry as well as possible, then pack full of carbolized wool, and proceed to fill as in an ordinary case. If convenient, I fill the cavity of decay with gutta percha and dismiss for a week. But it is not necessary.

The facility which the antiseptic method gives for utilizing firm roots, points that in the near future a great deal more will be done in the way of putting useful crowns on these. I believe Mr. Ladmore has done a good deal in this way, of which I cannot boast much, but I have had one in for about three years which answers very well. The one which I pass round I am about to fix for a patient.*

The treatment of teeth suffering with alveolar abscess or periostitis will not be entered upon, but before concluding I will just describe my method of preparing carbolized wool, with a case showing the reliability of it as a permanent filling for pulp cavities.

* The patient, for whom this gold crown was fixed, found it so useful that, two months later, she had another useless molar crowned in the same manner.

About seven years ago I filled the pulp cavity of the right upper cuspid of a lady with carbolized silk (which is practically the same), and the cavity of decay with gold and tinfoil.

About five years afterwards she came complaining of a good deal of pain about the region of this tooth, and as suspicion lay against the tooth, I removed the filling and the carbolized silk which came out as dry and aromatic as the day it was put in. The tooth was refilled and further search discovered the mischief in one of the bicuspids. I saw this lady a month ago and the tooth is firm and painless and without gum-boil.

I prepare the wool and silk in the following manner:

Black Resin........................................℥i

(Calvert's) liquid Carbolic Acid....................ʒiss

Melt the resin and add the carbolic acid.

Take some cotton wool and dip it in the mixture until it is saturated. Then wring out in muslin as much as possible and it is ready for use. Keep in a wide mouth stoppered bottle.

To prepare the silk. Take any floss silk you prefer and pass it through the melted mixture. I generally use the waxed silk of the depots.

